# Novel Antioxidant Collagen Peptides of Siberian Sturgeon (*Acipenser* *baerii*) Cartilages: The Preparation, Characterization, and Cytoprotection of H_2_O_2_-Damaged Human Umbilical Vein Endothelial Cells (HUVECs)

**DOI:** 10.3390/md20050325

**Published:** 2022-05-14

**Authors:** Yan Sheng, Yi-Ting Qiu, Yu-Mei Wang, Chang-Feng Chi, Bin Wang

**Affiliations:** 1 Zhejiang Provincial Engineering Technology Research Center of Marine Biomedical Products, School of Food and Pharmacy, Zhejiang Ocean University, Zhoushan 316022, China; 18223101235@163.com (Y.S.); qytdezh@icloud.com (Y.-T.Q.); wangym731@126.com (Y.-M.W.); 2 National and Provincial Joint Laboratory of Exploration and Utilization of Marine Aquatic Genetic Resources, National Engineering Research Center of Marine Facilities Aquaculture, School of Marine Science and Technology, Zhejiang Ocean University, Zhoushan 316022, China

**Keywords:** Siberian sturgeon (*Acipenser baerii*), cartilage, collagen peptide, antioxidant activity, cytoprotection

## Abstract

For making full use of aquatic by-products to produce high value-added products, Siberian sturgeon (*Acipenser baerii*) cartilages were degreased, mineralized, and separately hydrolyzed by five kinds of proteases. The collagen hydrolysate (SCH) generated by Alcalase showed the strongest 2,2-diphenyl-1-picrylhydrazyl radical (DPPH·) and hydroxide radical (HO·) scavenging activity. Subsequently, thirteen antioxidant peptides (SCP1-SCP3) were isolated from SCH, and they were identified as GPTGED, GEPGEQ, GPEGPAG, VPPQD, GLEDHA, GDRGAEG, PRGFRGPV, GEYGFE, GFIGFNG, PSVSLT, IELFPGLP, LRGEAGL, and RGEPGL with molecular weights of 574.55, 615.60, 583.60, 554.60, 640.64, 660.64, 885.04, 700.70, 710.79, 602.67, 942.12, 714.82, and 627.70 Da, respectively. GEYGFE, PSVSLT, and IELFPGLP showed the highest scavenging activity on DPPH· (EC_50_: 1.27, 1.05, and 1.38 mg/mL, respectively) and HO· (EC_50_: 1.16, 0.97, and 1.63 mg/mL, respectively), inhibiting capability of lipid peroxidation, and protective functions on H_2_O_2_-damaged plasmid DNA. More importantly, GEYGFE, PSVSLT, and IELFPGLP displayed significant cytoprotection on HUVECs against H_2_O_2_ injury by regulating the endogenous antioxidant enzymes of superoxide dismutase (SOD) and glutathione peroxidase (GSH-Px) to decrease the contents of reactive oxygen species (ROS) and malondialdehyde (MDA). Therefore, the research provided better technical assistance for a higher-value utilization of Siberian sturgeon cartilages and the thirteen isolated peptides—especially GEYGFE, PSVSLT, and IELFPGLP—which may serve as antioxidant additives for generating health-prone products to treat chronic diseases caused by oxidative stress.

## 1. Introduction

The balanced relationship between the endogenous antioxidant defense system and reactive oxygen species (ROS) will be broken under the toxic environment in the cells [1,2,3]. Excessive ROS can cause DNA mutation, enzyme inactivation, and membrane phospholipid oxidation, which further lead to oxidative stress, inducing cell necrosis or apoptosis, tissue injury, and pathologic transformations of the human body [4,5,6]. Such oxidative damage significantly increases the incidence of chronic diseases, including arthritis, hypertension, Alzheimer’s disease, diabetes, and cardiovascular disease [7,8,9,10]. Many antioxidant chemical compounds can play highly effective functions to prevent and to cure those diseases and to clear away excessive ROS in the human body [11,12]. However, synthetic antioxidants have shown a potential toxicity risk and their applications are strictly regulated [13,14]. Therefore, researchers are focusing their research interests on natural active molecules and their derivatives, such as flavonoids, triterpenoid, quinones, and alkaloids [12,15,16,17]. Remarkably, antioxidant peptide (AP) originated from food proteins, which captured worldwide interest because of their advantages in environmental protection and in sustainability, and their small molecular weight (MW) and low toxic side effects [1,9,12].

Collagen and its derivatives, including gelatin, hydrolysate, and peptide, are traditionally produced from animal bones and skins, and they have served as multifunctional ingredients applied in food, cosmetics, photographic, and pharmaceuticals products. The global market volume of gelatin/collagen is expected to exceed 650 kilo-tones, which is approximately 4 billion US dollars by 2024 [18,19,20,21]. However, those products generated from mammalian resources have aroused the wide concern of customers because of the increasing number of infectious diseases and dietary restriction in Islam, Judaism and Hinduism [22,23]. Therefore, collagen and its derivatives from fish by-products are considered to be ideal substitutes due to good bioactivity, high nutrition, weak antigenicity, excellent moisture retention, and good biocompatibility properties [18,24,25,26].

Recently, collagen peptides from aquatic organism drew great interest from the food, medicine, and cosmetics industries because of their multiple functions, including free radical scavenging activity, lipid peroxidation inhibition ability, cytoprotection, and ultraviolet damage protection [21,24,27,28]. For example, bioactive peptides from collagen hydrolysates of giant croaker swim bladders [29], sea cucumber [30], and redlip croaker scales [11] could significantly accelerate the proliferation of HUVECs, RAW264.7, and HepG2 cells, and protect them against the oxidative damage of H_2_O_2_ by increasing the activities of superoxide dismutase (SOD) catalase (CAT) and glutathione peroxidase (GSH-Px) and reducing the levels of ROS and malondialdehyde (MDA). Gelatin peptides from Pacific cod had a significant protective effect on ultraviolet-A (UVA) damaged cells and skins by up-regulating the levels of SOD, CAT, and GSH-Px [31,32,33]. Similarly, collagen peptides from silver carp skins showed a stronger beneficial effect than casein derived peptides and tea poly-phenols on alleviating the UV-caused unusual lesions of skin compositions and antioxidant indices in the serum and in the skins [34]. In addition, collagen peptides from the croceine croaker swim bladders showed a favorable anti-fatigue function in mice by increasing antioxidase activities to reduce ROS damage, enhancing the lactic dehydrogenase activity to get rid of excessive lactic acid to further alleviate the development of physical fatigue [35].

Sturgeon, belonging to the family Acipenseridae, is the common name of 27 kinds of cartilaginous fish, and its farmed production in China is approximately 4.4 million tons accounting for nearly 80% of world production [36,37]. In the receiving process of sturgeon eggs, cartilage, which accounts for 10% of the sturgeon’s weight, becomes a by-product. Therefore, active substances in sturgeon cartilage, such as chondroitin sulfate [38], collagen [39,40], and anti-inflammatory peptides [41], were studied constantly to replace shark cartilage, which is used in health and functional products. The Siberian sturgeon, *Acipenser baerii* Brandt, inhabits large Siberian rivers from the Ob to the Kolyma and Lake Baikal, and it is one of the important breeding varieties in China. In this experiment, antioxidant collagen peptides from the cartilage of the Siberian sturgeon (*A. baerii*) were prepared and identified. Moreover, their protective function on H_2_O_2_ injured HUVECs was evaluated.

## 2. Results and Discussion

### 2.1. Preparation of Collagen Hydrolysate of Siberian Sturgeon Cartilage (SCH)

**Figure 1:**
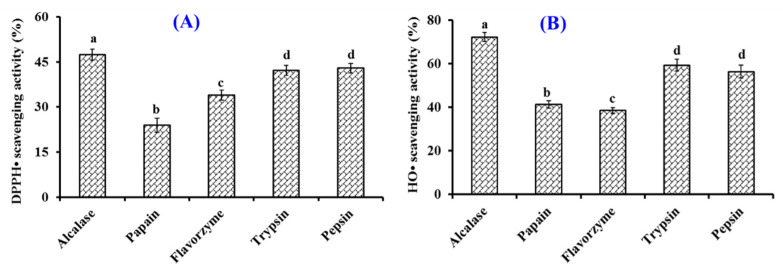
Effects of Alcalase, papain, pepsin, flavorzyme, and trypsin on radical scavenging activity of collagen hydrolysates from Siberian sturgeon (*Acipenser baerii*) cartilages. (**A**) 2,2-diphenyl-1-picrylhydrazyl radical (DPPH·) scavenging activity; (**B**) hydroxide radical (HO·) scavenging activity. All data are presented as the mean ± SD of triplicate results. ^a–d^ Values with different letters indicate significant difference (*p* < 0.05).

The effects of five kinds of proteases on the DPPH· and HO· scavenging rates of collagen hydrolysates of Siberian sturgeon cartilage are presented in Figure 1. At 10.0 mg/mL, the DPPH· and HO· scavenging rates of collagen hydrolysate generated by Alcalase were 47.43 ± 1.86% and 72.22 ± 2.11%, which were observably stronger than the rates of collagen hydrolysates produced using papain, flavorzyme, trypsin, and pepsin, respectively (*p* < 0.05). Compared with microbial fermentation, chemical degradation, and solvent extraction, enzymatic hydrolysis is one of the most popular and useful ways to generate bioactive hydrolysates from protein resources due to its easy manipulation, high efficiency, and eco-friendly features [1,42,43]. In addition, the specificity of protease is the very key property determining the MW, amino acid sequence, and bioactivity of the prepared hydrolysates because of their different cleavage sites [1,11]. In addition, multiple endonuclease enzymes, exonuclease enzymes, and their combinations are generally selected to degrade different proteins to generate active hydrolysates [1,9]. The present results supported the previous reports that the selectivity of enzymes significantly affected the peptide component and the bioactivities of prepared hydrolysates [1,14]. In consequence, the collagen hydrolysate of Siberian sturgeon cartilage prepared using Alcalase was named SCH and selected for further experimentation.

### 2.2. Purification of APs from SCH

#### 2.2.1. Fractionation of SCH by Ultrafiltration

**Figure 2:**
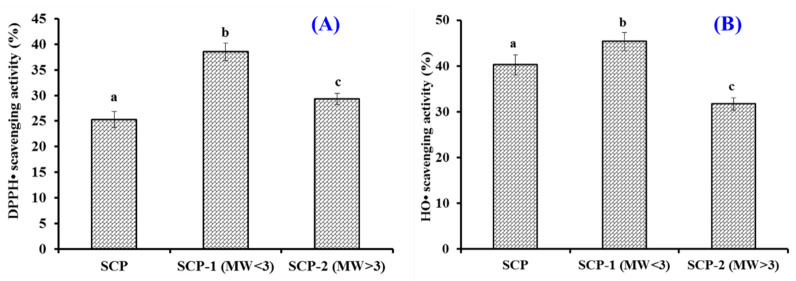
Radical scavenging activity of SCH and its two fractions by ultrafiltration. (**A**) DPPH· scavenging activity; (**B**): HO· scavenging activity. All data are presented as the mean ± SD of triplicate results. ^a–c^ Values with different letters indicate a significant difference (*p* < 0.05).

Using 3.0 kDa ultrafiltration membranes, SCH was fractionated into two peptide components (SCH-1 and SCH-2) and their radical scavenging rates are shown in Figure 2. At 5.0 mg/mL, the DPPH· and HO· scavenging rates of SCH-1 were 38.52 ± 1.69% and 45.37 ± 1.97%, which were significantly stronger than those activities of SCH and SCH-2 (*p* < 0.05). The changes of amino acid composition and MW could significantly modulate the bioactivity of peptides, and their average MWs could adversely affect the antioxidant capability of enzymatic hydrolysates [44,45]. The current result agreed well with the previous finding that peptide components with smaller MWs from skipjack roe [46,47,48], skate cartilage [49], *Bacillus amyloliquefaciens* [50], *Tolithes ruber* [51], croceine croaker muscle [13], and Tilapia skin [31] possessed the highest antioxidant activity. Then, SCH-1 was chosen for further purification.

#### 2.2.2. Gel Filtration Chromatography (GFC)

**Figure 3:**
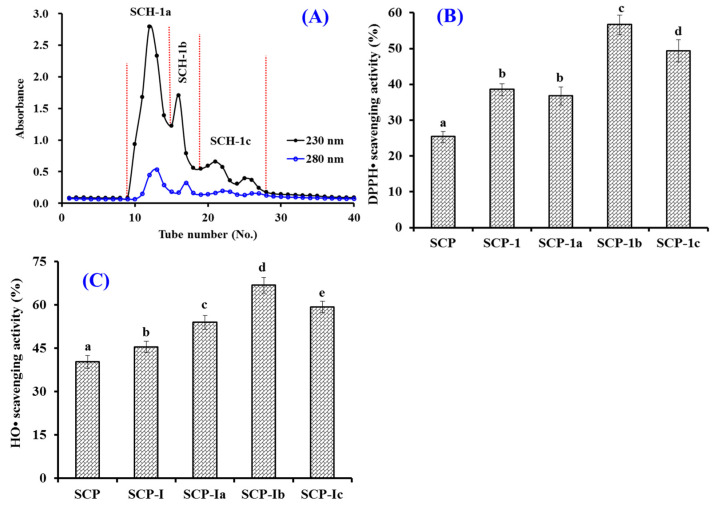
Chromatogram map of SCH-1 on a Sephadex G-25 column (**A**) and the scavenging activity of SCH-1 and its fractions (SCH-1a, SCH-1b, and SCH-1c) on DPPH· (**B**) and HO· (**C**). All data are presented as the mean ± SD of triplicate results. ^a–e^ Values with different letters indicate significant difference (*p* < 0.05).

Figure 3A showed that three peptide subfracitons (SCH-1a, SCH-1b, and SCH-1c) were isolated from SCH-1 based on their MWs. At 5.0 mg/mL, the DPPH· and HO· scavenging rates of SCH-1b were 56.64 ± 2.69% and 66.79 ± 2.65%, which were significantly higher than those of SCH, SCH-1, and other subfracitons (*p* < 0.05) (Figure 3B). As a kind of size exclusion chromatography, GFC is generally applied to either fractionate active ingredients or to remove an impurity with a particular size range from a complex mixture of components [1,9,52]. Therefore, GFC is frequently employed to isolate peptides with different MWs from marine protein hydrolysates [1,47,53]. In the experiment, the MW of SCH-1b was bigger than that of SCH-1c, but its radical scavenging rates were significantly higher than those of SCH-1c (*p* < 0.05), which suggested that the bioactivities of APs are not only influenced by MW but also amino acid composition and sequence [1,42].

#### 2.2.3. RP-HPLC Separation of SCH-1b

**Figure 4:**
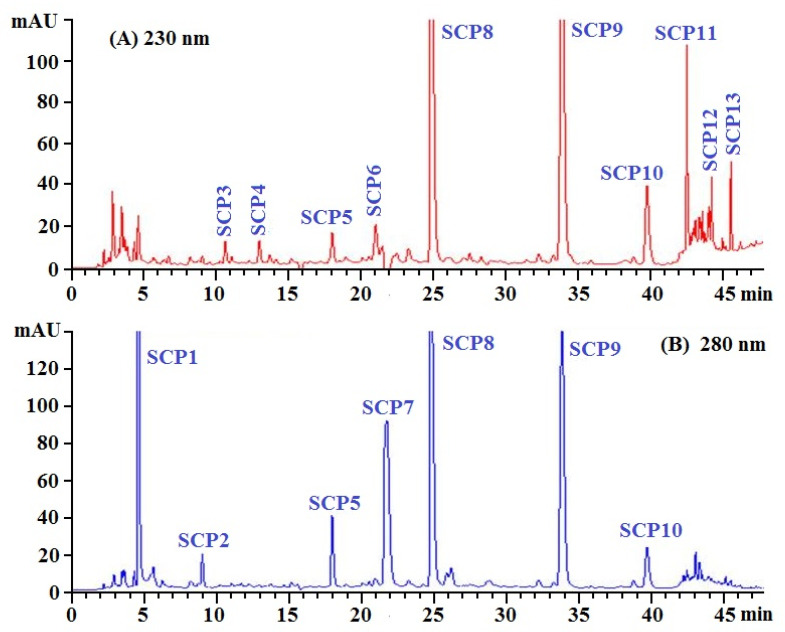
Elution profile of the subfraction (SCH-1b) by RP-HPLC using a linear gradient of acetonitrile (0.06% trifluoroacetic acid) at 230 nm (**A**) and 280 nm (**B**).

SCH-1 with high radical scavenging activity was further purified by RP-HPLC and its chromatogram is shown in Figure 4. On the chromatographic peaks of SCH-1, thirteen peptide peaks with retention times of 4.58 min (SCP1), 8.98 min (SCP2), 10.73 min (SCP3), 13.01 min (SCP4), 18.03 min (SCP5), 21.02 min (SCP6), 21.75 min (SCP7), 24.81 min (SCP8), 33.85 min (SCP9), 39.79 min (SCP10), 42.52 min (SCP11), 44.18 min (SCP12), and 45.62 min (SCP13), respectively, were purified from SCH-1b (Table 1). Based on the hydrophobic and the hydrophilic properties, RP-HPLC employing an ODSC18 column can effectively isolate APs with high purity from different protein hydrolysates of aquatic resources, such as croaker (*Otolithes ruber*) [51], tuna [46,54], red stingray [55], Pacific Cod [32,33], shortclub cuttlefish [56], *Euphausia superba* [57], and mackerel (*Scomber japonicus*) [58]. Then, thirteen peptides (SCP1 to SCP13) were corrected and lyophilized for further structure identification.

### 2.3. Determination of Amino Acid Sequences of Thirteen Isolated APs (SCP1 to SCP13)

Using a Protein Sequencer and an ESI/MS, the amino acid sequences and the MWs of thirteen isolated APs (SCP1 to SCP13) were determined and the results are shown in Table 1. The sequences of SCP1 to SCP13 were identified as Gly-Pro-Thr-Gly-Glu-Asp (GPTGED, SCP1), Gly-Glu-Pro-Gly-Glu-Gln (GEPGEQ, SCP2), Gly-Pro-Glu-Gly-Pro-Ala-Gly (GPEGPAG, SCP3), Val-Pro-Pro-Gln-Asp (VPPQD, SCP4), Gly-Leu-Glu-Asp-His-Ala (GLEDHA, SCP5), Gly-Asp-Arg-Gly-Ala-Glu-Gly (GDRGAEG, SCP6), Pro-Arg-Gly-Phe-Arg-Gly-Pro-Val (PRGFRGPV, SCP7), Gly-Glu-Tyr-Gly-Phe-Glu (GEYGFE, SCP8), Gly-Phe-Ile-Gly-Phe-Asn-Gly (GFIGFNG, SCP9), Pro-Ser-Val-Ser-Leu-Thr (PSVSLT, SCP10), Gly-Ile-Glu-Leu-Phe-Pro-Gly-Leu-Pro (GIELFPGLP, SCP11), Leu-Arg-Gly-Glu-Ala-Gly-Leu (LRGEAGL, SCP12), and Arg-Gly-Glu-Pro-Gly-Leu (RGEPGL, SCP13) with MWs of 574.55, 615.60, 583.60, 554.60, 640.64, 660.64, 885.04, 700.70, 710.79, 602.67, 942.12, 714.82, and 627.70 Da, respectively, and their determined MWs were well consistent with their theoretical mass (Table 1).

### 2.4. Antioxidant Activity of Thirteen Isolated APs (SCP1 to SCP13)

#### 2.4.1. Radical Scavenging Activity of Thirteen Isolated APs (SCP1 to SCP13)

**Figure 5:**
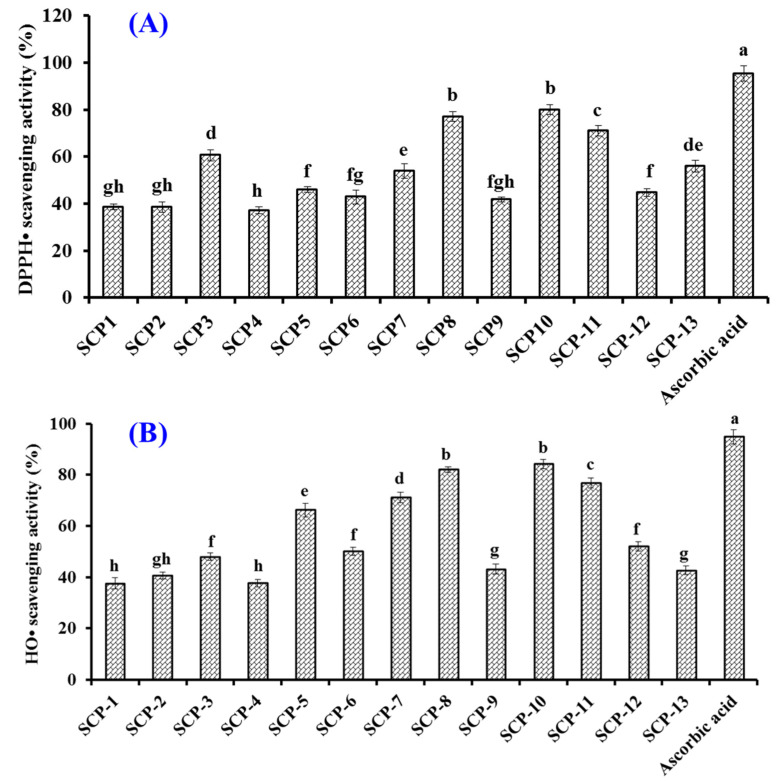
DPPH· (**A**) and HO· (**B**) scavenging rates of thirteen isolated APs (SCP1–SCP13) from collagen hydrolysate of Siberian sturgeon cartilages. All data are presented as the mean ± SD of triplicate results. ^a–h^ Values with different letters indicate significant difference (*p* < 0.05).

Figure 5A shows that the DPPH· scavenging rates of SCP8, SCP10, and SCP11 were 77.03 ± 2.08%, 80.09 ± 2.15%, and 71.1 ± 2.14%, respectively, which were significantly higher than those of ten other isolated collagen APs but still lower than that (95.37 ± 3.25%) of ascorbic acid. In addition, the half clearance concentrations (EC_50_ values) of SCP8, SCP10, and SCP11 were 1.27, 1.05, and 1.38 mg/mL, respectively, which were significantly less than those of APs from skipjack tuna milt (GRVPRV: 4.13 mg/mL; AQRPR 1.80 mg/mL) [59], loach (PSYV: 17.0 mg/mL) [60], Antarctic krill (NVPDM: 4.88 mg/mL; NGPDPRPSQQ: 7.05 mg/mL; TFPIYDPQ: 2.15 mg/mL) [61], and hairtail muscle (QNDER: 4.95 mg/mL) [62].

Figure 5B showed that the HO· scavenging rates of SCP8, SCP10, and SCP11 were 81.94 ± 1.05%, 84.11 ± 1.82%, and 76.78 ± 1.92%, respectively, which were significantly higher than those of ten other isolated collagen APs but still lower than that (94.84 ± 2.79%) of ascorbic acid. The EC_50_ values of SCP8, SCP10, and SCP11 on HO· were 1.16, 0.97, and 1.63 mg/mL, respectively, which were significantly less than those of APs from skipjack tuna milts (GRVPRV: 5.78 mg/mL; AQRPR 2.80 mg/mL) [59] and roes (SGE: 2.76 mg/mL; QAEP: 2.10 mg/mL) [48], miiuy croaker muscle (NFWWP: 2.39 mg/mL; YFLWP: 2.47 mg/mL) [63], Antarctic krill (NVPDM: 1.84 mg/mL; NWDDMRIVAV: 2.61 mg/mL) [61], *Misgurnus anguillicaudatus* (PSYV: 2.64 mg/mL) [60], and grass carp skin (VGGRP: 2.06 mg/mL; PYSFK: 2.28 mg/mL) [64]. The present results suggested that SCP8, SCP10, and SCP11 could effectively scavenge excess HO· to inhibit the oxidative stress in cells and biological tissues.

MW can significantly affect the antioxidant abilities of APs because a smaller size is beneficial to them in getting into cells or into tissues and playing their roles [50,65,66]. In the study, thirteen isolated APs (SCP1 to SCP13) range from pentapeptides to nonapeptides and their MWs range from 554.60 to 942.12 Da, respectively, which are very helpful for them to approach and to effectively scavenge excess free radicals.

Hydrophobic and aromatic amino acids, such as Leu, Ile, Tyr, Pro, and Phe, play key roles in the activity of APs. These two kinds of amino acids are able to improve the peptides’ solubility in lipids, which further facilitate the combination between APs and free radicals and promote the antioxidant capabilities of APs [1,9,50]. Leu, Thr, Ala, Ile, and Val were reported to play key roles in the antioxidant capabilities of HFGBPFH, ILGATIDNSK, GADIVA, and GAEGFIF, respectively [61,67,68]. Aromatic amino acids could restrain the extension of the radical-mediated peroxide domino effect by changing free radicals into more stable phenoxy radicals [63,69]. Pro residue in sequences of LDEPDPL and PHH was beneficial to their antioxidant activity because Pro residue could improve the flexibility of peptides and directly scavenge singlet oxygen by its pyrrolidine ring [59,70,71]. Therefore, Tyr and Phe in SCP8, Phe and Ile in SCP10, and Ile, Leu, Phe, and Pro in SCP11 should play key roles for their antioxidant activities.

Hydrophilic amino acids are the key factor for the scavenging abilities of APs on mental ions and hydroxide radicals [48]. Glu/Gln, Asp/Asn, and Lys residues had strong positive impacts on the antioxidant activities of QDHKA, AEHNH, LDEPDPLI, AEDKKLIQ, and NTDGSTDYGILQINSR [48,72,73]. Gly residue in WMGPY, EMGPA, GADIVA, and GAEGFIF could increase the flexibility of peptide skeleton and directly neutralize ROS by acting as a single hydrogen donor [25,74]. Therefore, Gly and Glu in SCP8, Gly and Asn in SCP10, and Gly and Glu in SCP11 were important to their antioxidant capabilities.

#### 2.4.2. Lipid Peroxidation Inhibition Ability

**Figure 6:**
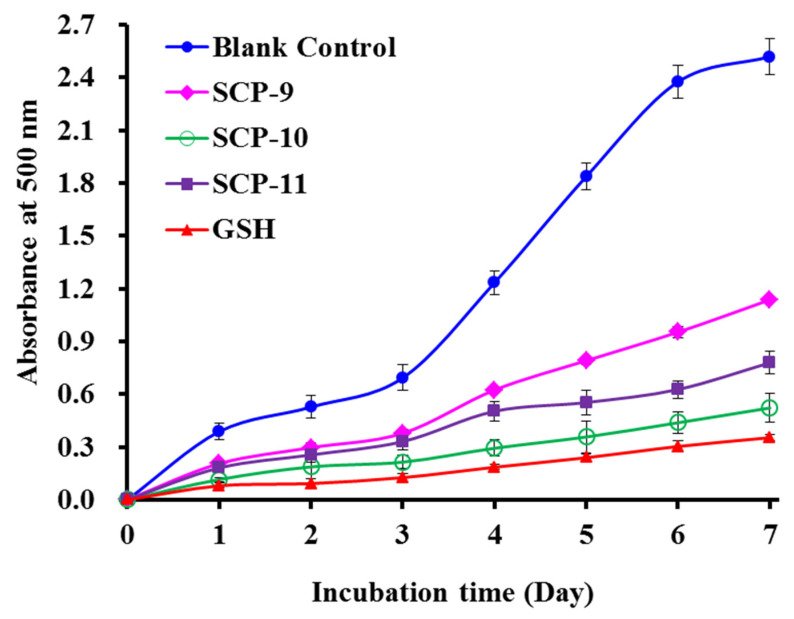
Lipid peroxidation inhibition capability of three isolated APs (SCP8, SCP10, and SCP11) from collagen hydrolysate of Siberian sturgeon cartilages. All data are presented as the mean ± SD of triplicate results.

Compared with the blank control group, the absorbance values of the SCP8, SCP10, and SCP11 groups at 500 nm were significantly decreased when the temperature was kept at 40 °C for 7 days in the linoleic acid system (Figure 6). More importantly, the inhibiting capabilities of SCP10 drew near the variation trend of glutathione (GSP). Lipid oxidation is a very complex chemical reaction, which is affected by multiple factors. Therefore, lipid peroxidation inhibition assay was generally applied to compare and to analyze the antioxidant abilities of peptides from marine protein resources, such as Antarctic krill [61], channel catfish [75], miiuy croaker [63], and croceine croaker [13]. These results suggested that SCP8, SCP10, and SCP11 have significant protective ability on unsaturated fatty acid against peroxidation.

#### 2.4.3. Protective Activity of SCP8, SCP10, and SCP11 against H_2_O_2_-damaged Plasmid DNA

**Figure 7:**
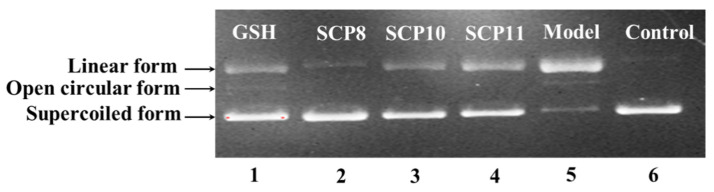
The protective effects of SCP8, SCP10, and SCP11 on the H_2_O_2_-damaged plasmid DNA (pBR322DNA). Lane 1, DNA + FeSO_4_ + H_2_O_2_ + GSH (200 μM); Lane 2, DNA + FeSO_4_ + H_2_O_2_ + SCP8 (200 μM); Lane 3, DNA + FeSO_4_ + H_2_O_2_ + SCP10 (200 μM); Lane 4, DNA + FeSO_4_ + H_2_O_2_ + SCP11 (200 μM); Lane 5, pBR322DNA + FeSO_4_ + H_2_O_2_; Lane 6, the native pBR322DNA.

The protective abilities of SCP8, SCP10, and SCP11 on plasmid DNA (pBR322DNA) against H_2_O_2_ damage were determined and presented in Figure 7. Plasmid DNA keeps the supercoiled (SC) form under normal conditions (Figure 7, lane 6), but the supercoiled (SC) form will translate into a relaxed open circular (OC) form when free radicals split one phosphodiester chain of pBR322 DNA. Moreover, the open circular (OC) form will turn into the linear (LIN) form when excess free radicals split the second breakage near the first splitting breakage. In this experiment, the plasmid DNA strands was split by HO·, produced from the chemical reaction of FeSO_4_ and H_2_O_2_, and converted into the OC and the LIN forms [61,76]. Lane 5 indicated that most of the SC forms of plasmid DNA were mutated to LIN forms, which suggested that the chemical reaction generated excessive HO·, which further broke the double-strand of pBR322 DNA. Lane 2 to Lane 4 displayed that the content of SC form of pBR322 DNA was obvious more than that of the model group (Lane 5), which suggested that SCP8, SCP10, and SCP11 have a remarkable effect on protecting plasmid DNA against oxidative damage by scavenging superfluous HO·, and this result agreed well with the previous finding that SCP8, SCP10, and SCP11 could effectively scavenge HO· to protect biomolecules. In addition, SCP8, SCP10, and SCP11 may serve as a radical scavenger in health products to prevent and to treat these degenerative diseases caused by free radicals.

#### 2.4.4. Cytoprotection of SCP8, SCP10, and SCP11 on H_2_O_2_-Induced HUVECs

##### Effects of H_2_O_2_, SCP8, SCP10, and SCP11 on the Viability of HUVECs

**Figure 8:**
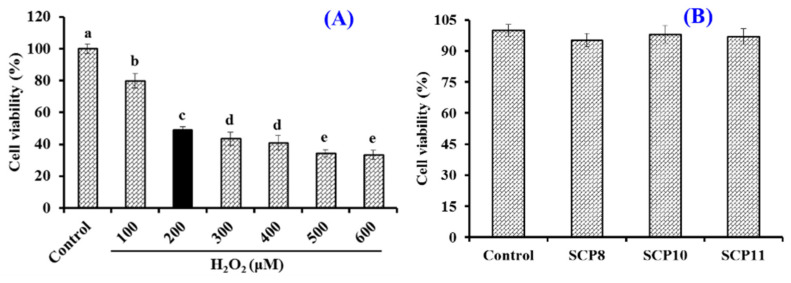
Effects of H_2_O_2_ concentration (**A**) and isolated peptides (SCP8, SCP10, and SCP11) (**B**) on the viability of HUVECs. All data are presented as the mean ± SD of triplicate results. ^a–e^ Values with different letters indicate significant difference (*p* < 0.05).

To establish the cell model of oxidative damage, HUVECs were treated with different concentrations of H_2_O_2_ (0~600 μM). Figure 8A indicated that the viability of HUVECs showed a significant downward trend at the H_2_O_2_ concentrations, which increased from 0 to 600 μM and dropped to 49.06 ± 1.96% at the concentration of 200 μM. Therefore, the H_2_O_2_ concentration of 200 μM was chosen to establish the cell model of oxidative damage [66].

The Effects of SCP8, SCP10, and SCP11 at 200 μM on the viability of HUVECs were studied by the MTT method and the data is shown in Figure 8B. No significant difference was found between the blank control and the peptide groups, which indicated that SCP8, SCP10, and SCP11 had no significant cytotoxicity to HUVECs. Therefore, the concentration of 200 μM was determined for the subsequent cytoprotection experiment of SCP8, SCP10, and SCP11.

##### Effect of SCP8, SCP10, and SCP11 on the Cell Viability and the ROS Level of H_2_O_2_-Injured HUVECs

**Figure 9:**
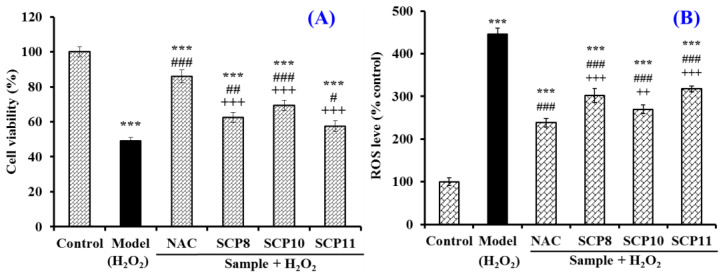
Effects of SCP8, SCP10, and SCP11 on the cell viability (**A**) and ROS level (**B**) of H_2_O_2_-injured HUVECs. N-Acetyl-L-Cysteine (NAC) was used as the positive control. All data are presented as the mean ± SD of triplicate results. *** *p* < 0.001 vs. blank group; ^###^ *p* < 0.001, ^##^ *p* < 0.01 and ^#^ *p* < 0.05 vs. model group; ^+++^ *p* < 0.001, ^++^ *p* < 0.01 vs. NAC + H_2_O_2_ group.

**Figure 10:**
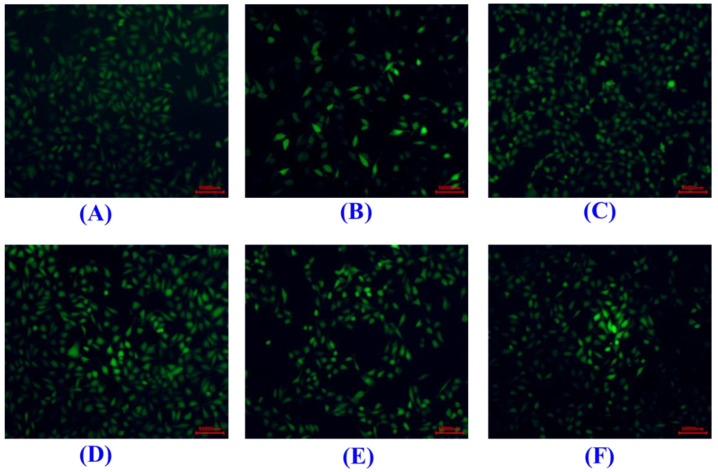
Determination of ROS contents in HUVECs by DCFH-DA staining. (**A**) Control; (**B**) H_2_O_2_-induced cell model; (**C**) Positive control (NAC); (**D**) SCP8; (**E**) SCP10; (**F**) SCP11. The scale bar was 50,000 nm.

As shown in Figure 9A, the HUVEC viability of the SCP10 group was 69.36 ± 2.97% at 200 μM, which was significantly higher than those of the model (49.06 ± 1.96%), SCP8 (62.4 ± 2.87%), and SCP11 (57.59 ± 3.21%) groups (*p* < 0.05), and it was lower than that of the positive control (86.03 ± 3.57%) (*p* < 0.001) (Figure 9A). Figure 9B and Figure 10 show the effects of SCP8, SCP10, and SCP11 on the ROS level of H_2_O_2_-injured HUVECs. The ROS levels of the SCP8, SCP10, and SCP11 groups were significantly decreased from 445.5 ± 14.57% to 302.2 ± 16.8%, 269.8 ± 11.5%, and 317.6 ± 6.4% for the control group, respectively (*p* < 0.001). These data indicated that SCP8, SCP10, and SCP11 could significantly scavenge ROS to protect HUVECs against H_2_O_2_ injury.

##### Effects of SCP8, SCP10, and SCP11 on the Levels of Antioxidases and MDA of H_2_O_2_-Injured HUVECs

**Figure 11:**
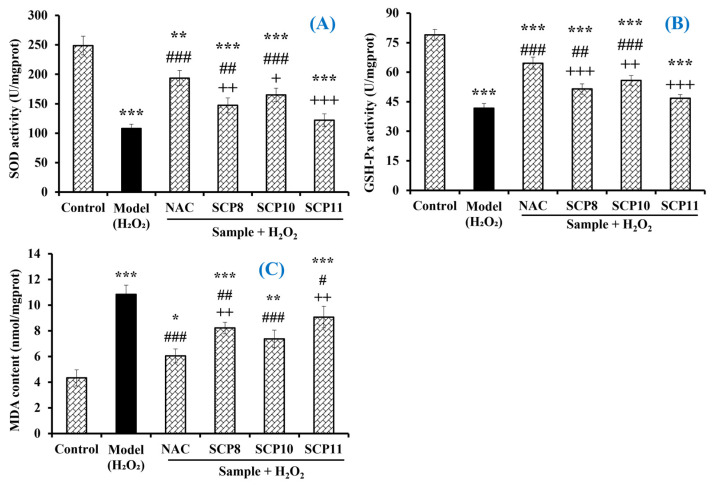
Effects of SCP8, SCP10, and SCP11 on the levels of SOD (**A**), GSH-Px (**B**), and MDA (**C**) in H_2_O_2_-injured HUVECs. All data are presented as the mean ± SD (*n* = 3). *** *p* < 0.001, ** *p* < 0.01 and * *p* < 0.05 vs. blank group; ^###^ *p* < 0.001, ^##^ *p* < 0.01, and ^#^ *p* < 0.05 vs. model group; ^+ + +^ *p* < 0.001 and ^++^ *p* < 0.01 and ^+^ *p* < 0.05 vs. NAC+H_2_O_2_ group.

As shown in Figure 11A, the activity of SOD in the SCP10 group was 165.1 ± 11.2 U/mg prot, which was significantly higher than those in the model (107.8 ± 7.3 U/mg prot) and the SCP8 (147.2 ± 12.6 U/mg prot) and SCP11 (121.9 ± 10.8 U/mg prot) groups (*p* < 0.001), respectively. Similarly, the activity of GSH-Px in the SCP10 group (55.77 ± 2.48 U/mg prot) was significantly higher than those in the model (41.74 ± 2.36 U/mg prot) and the SCP8 (51.46 ± 2.65 U/mg prot) and the SCP11 (46.8 ± 1.82 U/mg prot) groups (*p* < 0.001), respectively (Figure 11B). However, the activity of antioxidases in the SCP8, SCP10, and SCP11 groups was significantly lower than those in the positive control group (*p* < 0.05). In addition, SCP8, SCP10, and SCP11 could significantly reduce the MDA contents of H_2_O_2_-injured HUVECs. Compared with the model group (10.84 ± 0.72 nmol/mg prot), the MDA contents of the SCP8, SCP10, and SCP11 groups were gradually reduced to 8.22 ± 0.45, 7.37 ± 0.69, and 9.07 ± 0.84 nmol/mg prot at 200 μM, respectively (*p* < 0.05) (Figure 11C). Nonetheless, the MDA contents of the SCP8, SCP10, and SCP11 groups were significantly higher than that (6.05 ± 0.54 nmol/mg prot) of the positive control group.

In an abnormal environment, excess ROS generated in cells can induce DNA mutations, loss of protein structures, and lipid peroxidation of cell membrane [4,8,24]. Those oxidative stress states are closely linked to many chronic diseases, including neurodegenerative disorders, cardiovascular disease, diabetes mellitus, inflammation, etc. [10,12,52]. Therefore, excess ROS must be eliminated promptly and efficiently by endogenous antioxidant defense systems to decrease such oxidative damage [1,53]. Presently, some bioactive peptides show remarkable protection on cells and tissues by alleviating the oxidative and the inflammatory responses. For example, LCGEC could suppress the apoptosis of HaCaT cells by altering the Nrf2 pathway [47]. To decrease the contents of ROS and MDA, FWKVV, FMPLH, and FPYLRH could significantly up-regulate the levels of SOD and GSH-Px in H_2_O_2_-injured HUVECs [66,77]. By regulating the NF-κB/caspase pathways and enhancing antioxidase activities, EVSGPGLSPN could protect PC12 cells against H_2_O_2_-induced neurotoxicity [78].

In addition, small natural products have been identified as being capable of directly interacting with the Cys residues of Keap1 and thus resulting in the dissociation of Keap1 from Nrf2, which finally promotes Nrf2 nuclear accumulation and activates the Nrf2 pathway [79,80]. Moreover, a number of peptides have been identified to be capable of binding to Keap1, especially the Glu residues that form electrostatic interactions with R380, R415, and R483 and the Asp residue that forms an intramolecular interaction to stabilize the β-hairpin conformation of the structure [81]. The binding site of Keap1 in the Kelch domain can be divided into five subcysts, P1-P5, which can combine with the Neh2 domain of Nrf2 to promote its ubiquitination [82]. The five subcysts are P1 (Arg415, Ile461, Gly423, Phe478, Arg483, Ser508), P2 (Ser363, Arg380, Asn382, Asp422), P3 (Gly509, Ser555, Ala556, Gly571, Ser602, Gly603), P4 (Tyr525, Gln530, Tyr572), and P5 (Tyr334, Phe577), respectively. Wang et al. reported that the Glu residue of peptide EDYGA from the soft-shelled turtle could directly bind to the Arg415 residue on the Kelch domain of Keap1 to form a hydrogen bond [81]. Similarly, the Glu residues in an amino acid sequence of RDPEER from watermelon seed could combine with Asn382, Arg380, and Tyr334 on the Kelch domain of Keap1 to form hydrogen bonds [83]. Tonolo et al. found that the Ser residues in the amino acid sequence of APSFSDIPNPIGSENSE from fermented milk could bind to Arg415 and Ser363 residues on the Kelch domain of Keap1 to form a hydrogen bond to activate the Nrf2 pathway [84]. The Thr residues in the amino acid sequence of NTVPAKSCQAQPTTM could bind to the Ser602 residue in the Kelch domain of Keap1 to form a hydrogen bond [81]. Furthermore, Li et al. reported that the Thr residue of the peptide VTSALVGPR from the urechis unicinctus visceral could bind to Gly423 on the Kelch domain of Keap1 to form a hydrogen bond and to activate the Nrf2 pathway [85]. The EAMAPKHK from fermented rubbing cheese could regulate the Nrf2 pathway through its Pro residue combining with Asp422 on the Kelch domain of Keap1 to form a hydrogen bond [86]. In addition, the Gly residue in an amino acid sequence of PVLGPVR could combine with Ile461 on the Kelch domain of Keap1 to form a hydrogen bond [86]. Then, those amino acid residues in the amino acid sequences of APs occupy the active site of Nrf2 in the Kelch domain of Keap1, competitively inhibit Nrf2 binding, promote Nrf2 into the nucleus, further activate the Keap1/Nrf2 signal pathway, and protect cells from oxidative stress.

According to the introduced literature, we speculated that Gly and Glu in SCP8 (GEYGFE), Pro and Ser in SCP10 (PSVSLT), and Glu, Pro, and Gly in SCP11 (IELFPGLP) should play key roles in protecting HUVECs against H_2_O_2_ injury by regulating the endogenous antioxidant defense systems (Nrf2 pathway) to scavenge excess ROS, and their mechanism of action will be explored in our future studies.

## 3. Materials and Methods

### 3.1. Materials and Chemical Reagents

Cartilages of Siberian sturgeon (*A. baerii*) were kindly provided by Thousand Island Lake Sturgeon Technology Co., Ltd. (Hangzhou, China). HUVECs were purchased from the Cell Bank of Type Culture Collection of the Chinese Academy of Sciences (Shanghai, China). 3-[4,5-dimethylthiazol-2-yl]-2,5 diphenyl tetrazolium bromide (MTT), trypsin, Alcalase, NAC, DPPH, papain, and pepsin were purchased from Sigma-Aldrich Trading Co., Ltd. (Shanghai, China). Flavorzyme and Sephadex G-25 was purchased from Shanghai Source Poly Biological Technology Co., Ltd. (Shanghai, China). Collagen peptides of SCP1 to SC13 with a purity higher than 98% were synthesized in Shanghai Apeptide Co., Ltd. (Shanghai, China).

### 3.2. Preparation of Collagen Hydrolysate from Siberian Sturgeon Cartilages

The Siberian sturgeon cartilages were thawed, broken, homogenized, and degreased using the method described by Luo et al. [37]. In short, the cartilage was cut into approximately 0.5 cm^2^ pieces, homogenized, added into a NaOH solution (0.1 M) with a cartilage/solution ratio of 1:8 (*w*/*v*) and uninterruptedly stirred for 6 h, and the NaOH solution was substituted every three hours. Subsequently, the degreased cartilages were rinsed using cold tap water three times and demineralized using EDTA-2Na (0.5 M) with a cartilage/solution ratio of 1:8 (*w*/*v*) for two days, and the EDTA-2Na solution was changed every 12 h. The pretreated cartilage was rinsed using cold tap water three times.

Pretreated cartilages were suspended in a buffer solution to prepare the 10% (*w*/*v*) sample slurry. After that, the mixed solution was separately hydrolyzed for 6.5 h with 3.0% dose of Alcalase (pH 9.0, 50 °C), papain (pH 7.0, 50 °C), trypsin (pH 8.0, 37.0 °C), flavorzyme (pH 7.5, 45 °C), and pepsin (pH 2.0, 37.0 °C), respectively. The collagen hydrolysate solutions were put in a 95 °C water bath for 15 min to inactivate proteases, centrifuged at 6000× *g* for 20 min, dialyzed, and lyophilized. The activities of the prepared collagen hydrolysates were evaluated using DPPH· and HO· scavenging assays [45]. Then, the collagen hydrolysate produced using Alcalase revealed the maximum activity among the five hydrolysates, and it was named SCH.

### 3.3. Purification of APs from SCH

**Figure 12:**
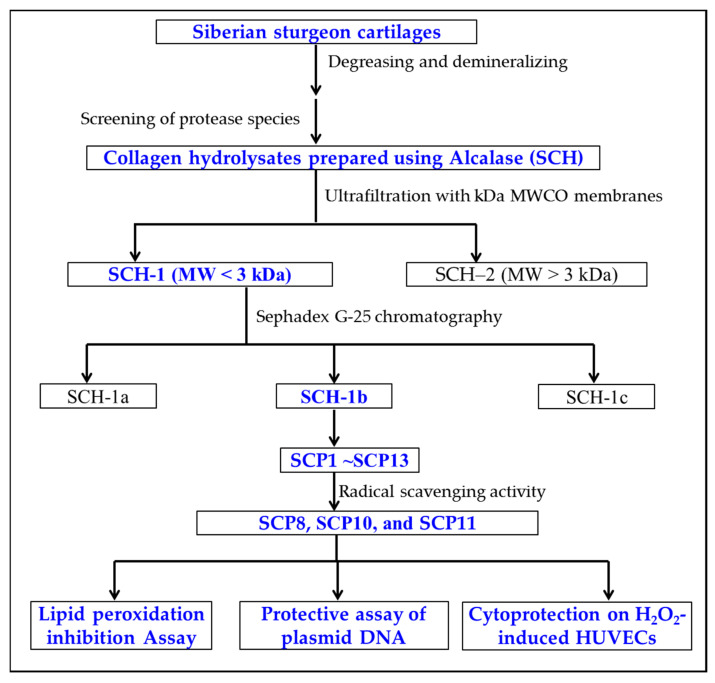
The flow chart of preparation and activity evaluation of APs from collagen hydrolysate (SCH) of Siberian sturgeon cartilages.

APs were prepared from SCH according to the following designed isolated process (Figure 12).

The SCH solution was fractionated using a 3 kDa MW cut-off ultrafiltration membrane and two resulting components, defined as SCH-1 (MW < 3 kDa), and SCH-2 (MW > 3 kDa) were collected, dialyzed, freeze-dried, and their radical scavenging activity was detected.

A total of 10 mL of SCH-1 solutions (50.0 mg/mL) were injected into the chromatography column of Sephadex G-25 (2.6 cm × 150 cm) and washed out by phosphate buffer solution (PBS, pH 7.2), with a flow rate of 1.0 mL/min. The effluent solution was collected every 2 min and measured at 230 and 280 nm. Finally, three peptide components (SCH-1a, SCH-1b, and SCH-1c) were enriched, desalted, freeze-dried, and their radical scavenging activity was detected.

The SCH-1b (20 μL, 100.0 μg/mL) was pre-treated with a 0.22 μm microporous membrane and purified by a HPLC column of Waters Symmetry C18 (4.6 × 250 mm, 5 µm) using a gradient of acetonitrile containing 0.06% trifluoroacetic acid. The sample was isolated with a flow velocity of 0.8 mL/min and monitored at 230 and 280 nm. In the end, thirteen APs (SCP1 to SCP13) were purified from SCH-1b on the basis of the chromatographic peaks.

### 3.4. Analysis of Sequences and MWs of Thirteen APs (SCP1 to SCP13)

The N-terminal amino-acid sequences of thirteen APs (SCP1 to SCP13) were determined by the Edman degradation method using an Applied Biosystems 494 protein sequencer (Foster City, CA, USA). The MWs of thirteen APs (SCP1 to SCP13) were measured by a Q-TOF MS coupled to an electrospray ionization (ESI) source.

### 3.5. Radical Scavenging, Lipid Peroxidation Inhibition, and Plasmid DNA Protective Assays

#### 3.5.1. Radical Scavenging Assays

The DPPH· and the HO· scavenging assays were performed on the previous methods, and the EC_50_ value was set as the AP dose, resulting in a 50% decrease of the initial radical concentration [14,45].

##### DPPH· Scavenging Activity

Two milliliters of samples consisting of distilled water and different concentrations of the analytes were placed in cuvettes, and 500 μL of an ethanolic solution of DPPH (0.02%) and 1.0 mL of ethanol were added. A control sample containing the DPPH solution without the sample was also prepared. In the blank, the DPPH solution was substituted with ethanol. The antioxidant activity of the sample was evaluated using the inhibition percentage of the DPPH radical with the following equation:

DPPH radical scavenging activity (%) = (A0 + A′ − A)/A0 × 100%(1)

where A is the absorbance rate of the sample, A0 is the control group absorbance, and A′ is the blank absorbance.

##### HO· Scavenging Activity

A total of 1.0 mL of a 1.87 mM 1,10-phenanthroline solution and 2.0 mL of the sample were added to a screw-capped tube and mixed. Then, 1.0 mL of a FeSO_4_·7H_2_O solution (1.87 mM) was added to the mixture. The reaction was initiated by adding 1.0 mL of H_2_O_2_ (0.03%, *v*/*v*). After incubating at 37 °C for 60 min in a water bath, the absorbance of the reaction mixture was measured at 536 nm against a reagent blank. The reaction mixture without any antioxidant was used as the negative control, and a mixture without H_2_O_2_ was used as the blank. The hydroxyl radical scavenging activity (HRSA) was calculated using the following formula:

HRSA (%) = [(As − An)/(Ab − An)] × 100%(2)

where As, An, and Ab are the absorbance values determined at 536 nm of the sample, negative control, and blank after the reaction, respectively.

#### 3.5.2. Lipid Peroxidation Inhibition Assay

Lipid peroxidation inhibition assays were operated on the reported methods [11,14]. Briefly, a sample (5.0 mg) was dissolved in 10 mL of 50 mM PBS (pH 7.0) and added to 0.13 mL of a solution of linoleic acid and 10 mL of 99.5% ethanol. Then, the total volume was adjusted to 25 mL with deionized water. The mixture was incubated in a conical flask with a screw cap at 40 °C in a dark room, and the degree of oxidation was evaluated by measuring ferric thiocyanate values. The reaction solution (100 μL) incubated in the linoleic acid model system was mixed with 4.7 mL of 75% ethanol, 0.1 mL of 30% ammonium thiocyanate, and 0.1 mL of 20 mM ferrous chloride solution in 3.5% HCl. After 3 min, the thiocyanate value was measured at 500 nm, following color development with FeCl_2_ and thiocyanate at different intervals during the incubation period at 40 °C.

#### 3.5.3. Protective Assay on Plasmid DNA

The protective effects of SCP8, SCP10, and SCP11, on supercoiled plasmid DNA (pBR322) were measured using the previous method [11]. In brief, 15 μL of reaction mixtures containing 5 μL of PBS (10 mM, pH 7.4), 2 μL of FeSO_4_ (1.0 mM), 1μL of pBR322 (0.5 μg), 5 μL of the peptide (SCP8, SCP10, or SCP11, respectively), and 2 μL of H_2_O_2_ (1.0 mM) were incubated at 37 °C. After 0.5 h incubation, the reaction was terminated by adding 2 μL of a loading buffer containing glycerol (50%, *v*/*v*), ethylenediaminetetraacetic acid (40 mM), and bromophenol blue (0.05%). The resulted reaction mixtures were subsequently electrophoresed on 1% agarose gel containing 0.5 μg/mL EtBr for 50 min (60 V), and the DNA in the agarose gel was photographed under ultraviolet light.

### 3.6. Protective Function of SCP8, SCP10, and SCP11 on H_2_O_2_-Injured HUVECs

#### 3.6.1. Cell Culture and Viability Determination

The HUVECs were cultured according to the described method by Cai et al. [66] and Wang et al. [77]. In brief, HUVECs with the density of 1.0 × 10^5^ cells/well were seeded into a 96-well plate containing 100 μL of culture media. After incubated for 24 h, 20 μL of SCP8, SCP10, and SCP11 solutions dissolved in the DMEM medium were separately added in the sample groups with the final concentration of 200 μg/mL. In addition, peptide was substituted by PBS (pH 7.2) in the control group. After incubated for 24 h, 20 μL of MTT was added into the plate and OD490 nm was measured after 4 h. The cell viability was calculated on the basis of the following formula:

Cell viability (%) = (OD_sample_/OD_control_)×100. (3)

#### 3.6.2. Protection of SCP8, SCP10, and SCP11 on H_2_O_2_-Injured HUVECs

HUVECs with the density of 1.0 × 10^5^ cells/well were seeded into a 96-well plate containing 100 μL of culture media. After 24 h, the supernatant in the HUVECs wells was aspirated and H_2_O_2_ was added, and its final concentrations, respectively, reached 0, 100, 200, 300, 400, 500, and 600 μM. After 24 h, cell viability was determined according to the above method and the H_2_O_2_ concentration that induced cell viability by approximately 50% was chosen to establish the oxidative damage model of HUVECs [66,77].

After culturing for 24 h, the supernatant in the HUVECs wells was wiped off. Subsequently, 100 µL of the peptide samples at the final concentrations of 200 µM were joined in the protection groups. After 8 h, the peptide sample was cleared and H_2_O_2_ at 200 µM was put in the model and the peptide sample groups and then treated for 24 h. A total of 100 µL of NAC (1.5 mM) was used as the positive control group. The blank control group used 20 μL PBS instead of the peptide solution.

#### 3.6.3. Determination of ROS, MDA, and Antioxidases

The levels of ROS in the blank control, model, and sample groups were measured on the reported method and expressed as a percentage of the of blank control [66].

The activity of SOD and GSH-Px and the content of MDA were measured using assay kits in accordance with the protocols of the Nanjing Jiancheng Bioengineering Institute Co., Ltd. (Nanjing, China), and the levels of SOD and GSH-Px were indicated as U/mg prot.

### 3.7. Statistical Analysis

The data are expressed as the mean ± standard deviation (SD, *n* = 3). An ANOVA test was used to analyze the differences between the means of each group, using SPSS 19.0 (Statistical Program for Social Sciences, SPSS Corporation, Chicago, IL, USA). A Duncan’s test was used to determine the significance between different groups (*p* < 0.05, *p* < 0.01, or *p* < 0.001).

## 4. Conclusions

In the study, thirteen APs were isolated from the collagen hydrolysate of Siberian sturgeon cartilages produced using Alcalase and identified as GPTGED, GEPGEQ, GPEGPAG, VPPQD, GLEDHA, GDRGAEG, PRGFRGPV, GEYGFE, GFIGFNG, PSVSLT, IELFPGLP, LRGEAGL, and RGEPGL, respectively. Among them, GEYGFE, PSVSLT, and IELFPGLP showed the highest radical scavenging activity, lipid peroxidation inhibiting capability, and protection on H_2_O_2_-injured HUVECs and on plasmid DNA. Therefore, this research provides free technical support for higher-valued utilizing fish by-products. More importantly, thirteen isolated collagen APs, especially GEYGFE, PSVSLT, and IELFPGLP, may act as antioxidant additives for generating health products to treat chronic diseases caused by oxidative stress. Moreover, the antioxidant mechanism of GEYGFE, PSVSLT, and IELFPGLP will be systematically researched in our follow-up study.

## Figures and Tables

**Table 1:** Retention time, amino acid sequences, and molecular mass of thirteen APs (SCP1- SCP13) from collagen hydrolysate of Siberian sturgeon cartilage.

	Retention Time (min)	Amino Acid Sequence	Determined Mass/Theoretical Mass (Da)
SCP1	4.58	GPTGED	574.55/574.54
SCP2	8.98	GEPGEQ	615.60/615.59
SCP3	10.73	GPEGPAG	583.60/583.59
SCP4	13.01	VPPQD	554.60/554.59
SCP5	18.03	GLEDHA	640.64/640.65
SCP6	21.02	GDRGAEG	660.64/660.63
SCP7	21.75	PRGFRGPV	885.04/885.02
SCP8	24.81	GEYGFE	700.70/700.69
SCP9	33.85	GFIGFNG	710.79/710.78
SCP10	39.79	PSVSLT	602.67/602.68
SCP11	42.52	GIELFPGLP	942.12/942.11
SCP12	44.18	LRGEAGL	714.82/714.81
SCP13	45.62	RGEPGL	627.70/627.69

## Data Availability

Data are contained within the article.

## Abbreviations

DPPH·, 2,2-diphenyl-1-picrylhydrazyl radical; HO·, hydroxide radical; ROS, reactive oxygen species; AP, antioxidant peptide; MW, molecular weight; SCH, Collagen hydrolysate of Siberian Sturgeon Cartilage; GFC, Gel Filtration Chromatography; SCP1, Gly-Pro-Thr-Gly-Glu-Asp (GPTGED); SCP2, Gly-Glu-Pro-Gly-Glu-Gln (GEPGEQ); SCP3, Gly-Pro-Glu-Gly-Pro-Ala-Gly (GPEGPAG); SCP4, Val-Pro-Pro-Gln-Asp (VPPQD); SCP5, Gly-Leu-Glu-Asp-His-Ala (GLEDHA); SCP6, Gly-Asp-Arg-Gly-Ala-Glu-Gly (GDRGAEG) SCP7, Pro-Arg-Gly-Phe-Arg-Gly-Pro-Val (PRGFRGPV); SCP8, Gly-Glu-Tyr-Gly-Phe-Glu (GEYGFE); SCP9, Gly-Phe-Ile-Gly-Phe-Asn-Gly (GFIGFNG); SCP10, Pro-Ser-Val-Ser-Leu-Thr (PSVSLT); SCP11, Gly-Ile-Glu-Leu-Phe-Pro-Gly-Leu-Pro (GIELFPGLP); SCP12, Leu-Arg-Gly-Glu-Ala-Gly-Leu (LRGEAGL); SCP13, Arg-Gly-Glu-Pro-Gly-Leu (RGEPGL); GSP, glutathione; SC, supercoiled; OC, open circular; LIN, linear; HUVECs, Human umbilical vein endothelial cells; MTT, 3-(4,5-Dimethylthiazol-2yl)-2,5-diphenyltetrazolium bromide.
